# Padua Score and D-dimer for Pulmonary Embolism Exclusion in the Elderly

**DOI:** 10.5041/RMMJ.10548

**Published:** 2025-07-31

**Authors:** Ramon Cohen, Shay Nemet, Marina A. Fradkin, Tal Schiller, Alena Kirzhner, Haitham Abu Khadija, Meital Deitch, Daniel Elbirt

**Affiliations:** 1 Faculty of Medicine, Hebrew University of Jerusalem, Jerusalem, Israel; 2 Department of Internal Medicine B, Kaplan Medical Center, Rehovot, Israel; 3 Department of Clinical Immunology Allergy and AIDS, Kaplan Medical Center, Rehovot, Israel; 4 Department of Radiology, Rabin Medical Center, Petah Tikva, Israel; 5 Department of Diabetes, Endocrinology and Metabolism, Kaplan Medical Center, Rehovot, Israel; 6 Department of Internal Medicine A, Kaplan Medical Center, Rehovot, Israel; 7 Heart Center, Kaplan Medical Center, Rehovot, Israel; 8 Intensive Care Unit, Kaplan Medical Center, Rehovot, Israel

**Keywords:** D-dimer, elderly, inflammation, Padua, pulmonary embolism

## Abstract

**Purpose:**

This study was aimed at identifying biomarkers that could help exclude pulmonary embolism (PE) in patients aged 65 years and older, considering age-related challenges such as atypical clinical presentations and the presence of comorbidities.

**Methods:**

This single-center cohort study retrospectively collected data on 28 potential markers from patients aged 65 years and older who underwent computed tomography scans for PE diagnosis in emergency or internal wards over a 2.5-year period.

**Results:**

The study included 157 patients after exclusions, with 35 diagnosed with PE. Patients with PE exhibited higher D-dimer levels, lower platelet counts, and higher Padua scores. Six markers were selected based on likelihood ratio, each with an area under the curve above 0.7 and *P*-value below 0.05. Multiplying D-dimer levels with the Padua score (PaDd) improved specificity from 9% to 32% while maintaining 100% sensitivity in identifying PE. Further refinement by incorporating activated partial thromboplastin time (aPTT) into the Padua score multiplied by D-dimer (PaDd/aPTT) resulted in improved sensitivity and specificity.

**Conclusion:**

The Padua score multiplied by D-dimer is a simple yet effective tool that enhances specificity while maintaining high sensitivity, potentially reducing computed tomography utilization in elderly patients. Prospective, multicenter studies are needed to validate these findings and integrate them into routine clinical practice.

## INTRODUCTION

Pulmonary embolism (PE) is a life-threatening condition caused by the sudden occlusion of one or more pulmonary arteries, usually due to a thrombus originating from deep vein thrombosis. Prompt and accurate diagnosis is crucial to initiate appropriate treatment and prevent complications. However, diagnostic challenges in specific populations, particularly the elderly, contribute to delays in treatment and increased morbidity and mortality.^1^

The diagnostic approach for PE typically involves clinical evaluation, imaging studies, and laboratory tests, including D-dimer measurement, which assists in risk stratification.^1^ Contrast-enhanced computed tomography (CT) remains the gold standard for PE diagnosis. However, its use carries inherent risks, including allergic reactions, nephrotoxicity, and patient-related limitations such as claustrophobia.^2^

### Pulmonary Embolism in the Elderly

Current diagnostic strategies often rely on clinical prediction rules, such as the Wells criteria, in conjunction with D-dimer testing, particularly in cases with low or moderate pretest probability.^3^ Diagnosing PE in elderly patients presents specific challenges due to age-related physiological changes, comorbidities, and atypical clinical presentations. Unlike younger individuals, who often report acute dyspnea and chest pain, elderly patients may exhibit non-specific symptoms such as confusion, fatigue, dizziness, or a general decline in functional status. Additionally, the presence of multiple chronic conditions—such as cardiovascular disease, hypertension, diabetes, and chronic obstructive pulmonary disease—can obscure the clinical picture, complicating diagnostic accuracy.^4^ Diagnosing PE in the oldest-old (≥85 years) is particularly complex due to multiple comorbidities, cognitive impairment, and atypical presentations.^5^,^6^ Traditional diagnostic criteria may be less reliable in this population.^7^,^8^ Established PE assessment tools, such as the Wells and Geneva scores, have demonstrated reduced accuracy in elderly patients.^9^ Furthermore, reliance of the Wells score on the physician’s judgment compromises standardization.^10^

### Biomarkers and Prognostic Scores Used in Pulmonary Embolism Evaluation

D-dimer is a widely used biomarker in the evaluation of PE, deep venous thrombosis and disseminated intravascular coagulation.^11^ However, while an elevated D-dimer level suggests ongoing fibrinolysis, it is not specific to thromboembolic events. Various inflammatory and infectious conditions can also increase D-dimer levels due to the interplay between inflammation and coagulation.^12^ This lack of specificity is particularly problematic in elderly patients, where baseline D-dimer levels tend to be higher, complicating its interpretation as a rule-out test. The age-adjusted D-dimer threshold has been proposed to improve specificity in older populations; however, its clinical utility remains debated, with no clear consensus on its application.^13^–^15^ Beyond D-dimer, other biomarkers have been explored for their potential role in PE diagnosis and prognostication. The neutrophil-to-lymphocyte ratio has been identified as an indicator of systemic inflammation and has been associated with increased mortality risk in acute PE.^16^ Similarly, the platelet-to-lymphocyte ratio has been studied as a marker of inflammation and thrombogenicity, with potential prognostic implications.^17^,^18^

The Padua Prediction Score is a validated tool designed to assess venous thromboembolism risk in hospitalized patients.^19^ Its potential utility in outpatient PE risk assessment remains under investigation.^20^

Despite extensive research, no biomarker has yet demonstrated sufficient diagnostic accuracy to significantly reduce reliance on imaging in this population.^21^

We conducted a literature search to identify biomarkers and clinical scores that could improve specificity while maintaining high sensitivity. The literature highlighted several key findings: D-dimer remains the most sensitive marker for PE but lacks specificity in elderly patients. The Padua score is widely used for venous thromboembolism risk assessment but has not been fully explored for PE diagnosis. Inflammatory markers and coagulation parameters have been proposed as adjuncts to D-dimer but lack consistent validation.

Based on the literature, we selected test combinations that offer the best potential to improve specificity while maintaining 100% sensitivity. As mentioned briefly above, the Wells criteria play a central role in guiding the clinical management of pulmonary embolism. For example, the Padua score × D-dimer (PaDd) score was chosen because it integrates both clinical risk and laboratory findings. Additional biomarkers (platelet count, activated partial thromboplastin time [aPTT]) were included based on their reported associations with thrombotic risk and their potential to refine diagnostic accuracy.

This study was aimed at evaluating the performance of specific diagnostic indicators in identifying elderly patients with PE. By refining risk stratification strategies, we sought to improve diagnostic accuracy and reduce unnecessary CTs in this vulnerable population.

## PATIENTS AND METHODS

This single-center retrospective study was approved by the Institutional Review Board and conducted on September 9, 2023, using electronic medical records from patients hospitalized between January 1, 2021, and June 1, 2023.

### Inclusion and Exclusion Criteria

**Figure 1:**
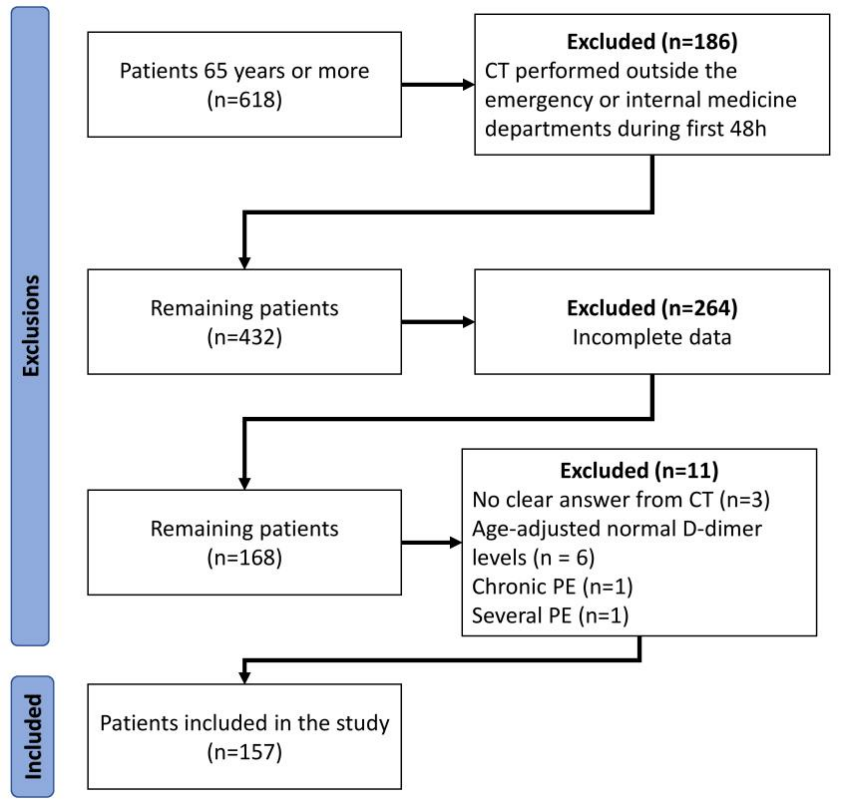
Flow Diagram Showing Application of Study Exclusion Criteria. CT, computed tomography; PE, pulmonary embolism.

Patients aged 65 years or older who underwent a CT for suspected PE were included. Patients diagnosed with sub-segmental PE on CT were considered negative for PE (Figure 1). The exclusion criteria are detailed in Table 1.

**Table 1:** Patient Exclusion Criteria.

Exclusion Criteria
Inconclusive CT results (“suspicion of pulmonary embolism”)

Chronic PE

Incomplete clinical data

Underwent multiple CTs to rule out PE during hospitalization

Age-adjusted normal D-dimer levels tenfold below patient’s age *
CT performed outside of the emergency or internal medicine department within first 48 h of hospitalization
No biomarker or D-dimer blood sample acquired during the 48 hours before CT
Padua score not calculated on admission to ward†

CT, computed tomography; PE, pulmonary embolism.

* The literature has accepted this threshold for older adults.^13^

† Calculation of Padua score is a standard

### Data Collection

Demographic data and laboratory results were collected, including albumin, calcium, international normalized ratio, prothrombin time, aPTT, mean platelet volume (MPV), D-dimer, C-reactive protein (CRP), fibrinogen, neutrophils, lymphocytes, and platelets. Blood samples were drawn within 48 hours prior to CT. If multiple measurements were available for a patient, only the first recorded value was used for analysis.

The Padua Prediction Score was calculated on the first day of hospitalization in the internal medicine ward. The score component for “previous venous thromboembolism” was assigned only if it had occurred prior to the current hospitalization.

### Study Design

Based on literature review and study hypotheses, the following biomarker combinations were analyzed:

- D-dimer-to-fibrinogen ratio^22^
- Platelet-to-lymphocyte ratio^23^
- Neutrophil-to-lymphocyte ratio^23^
- Immune-inflammation index: neutrophil × platelet-to-lymphocyte ratio^24^
- D-dimer-to-CRP ratio^25^
- Fibrinogen-to-CRP ratio^26^
- Fibrinogen-to-albumin ratio^27^
- Platelet × D-dimer-to-lymphocyte ratio
- Padua × D-dimer (PaDd)
- D-dimer-to-aPTT ratio
- D-dimer × Padua-to-CRP ratio
- D-dimer × Padua-to-platelet (PaDd/PLT) ratio
- D-dimer × Padua-to-aPTT (PaDd/PTT) ratio

### Statistical Analysis

Patients were categorized into two groups based on CT results: PE-positive and PE-negative. Univariate binary logistic regression was performed to identify biomarkers associated with PE, as this method allows for a straightforward evaluation of individual predictors. Variables with a likelihood ratio test *P*-value <0.05 were considered statistically significant. Odds ratios and 95% confidence intervals were calculated for significant variables.

To assess the discriminatory power of selected variables, receiver operating characteristic curve analysis was conducted. Variables with an area under the curve >0.7 were considered for further analysis (*P*<0.05). Specificity was evaluated at a sensitivity of 100% to ensure no PE cases were missed. The chi-square test or Fisher’s exact test was used for *P*-value calculations. All analyses were performed using Python (Version 3.11-Python Software Foundation, Wilmington, DE, USA).

## RESULTS

During the study period, 618 patients aged 65 years or older had a CT for PE diagnosis. After application of the exclusion criteria, a total of 157 patients were included in the final analysis (Figure 1).

Patients were further categorized as PE-positive or PE-negative based on their CT results. Table 2 summarizes the patient characteristics. Individuals with PE tended to have higher D-dimer levels, lower platelet counts, and a higher Padua score compared to those without PE. Additionally, significant differences in coagulation factors were shown, such as in aPTT. However, no significant differences were observed in other biomarkers and inflammatory markers between the two groups.

**Table 2:** Comparison of Population Characteristics, With and Without Pulmonary Embolism.

Characteristic	No PE (*n*=122)	PE (*n*=35)	*P*-value
Sex, *n* (%)			
Male	52 (43%)	12 (34%)	–
Female	90 (57%)	23 (66%)	–
Medications, *n* (%)			
Aspirin	31 (25%)	15 (42%)	0.06
Clopidogrel	13 (10%)	2 (6%)	0.52
Warfarin	1 (<1%)	1 (3%)	0.39
NOAC/LMWH	9 (7%)	1 (3%)	0.45
Age (years), mean (SD)	77.3 (8.8)	79.9 (8.6)	0.10
D-dimer (μg/mL), mean (SD)	6.73 (11.72)	12.63 (13.9)	0.02
Fibrinogen (mg/dL), mean (SD)	462 (182)	429 (134)	0.25
Platelet (1000/μL), mean (SD)	261 (156)	204 (84)	<0.01
Neutrophil abs (1000/μL), mean (SD)	7.30 (4.50)	7.60 (2.51)	0.60
Lymphocyte abs (1000/μL), mean (SD)	1.28 (0.76)	1.55 (1.17)	0.19
CRP (mg/dL), mean (SD)	8.10 (8.8*)	7.83 (5.96)	0.83
Albumin (g/dL), mean (SD)	3.40 (0.40)	3.30 (0.50)	0.48
Calcium (mg/dL), mean (SD)	8.7 (0.70)	8.84 (0.84)	0.34
INR, mean (SD)	1.18 (0.92)	1.07 (0.12)	0.23
PT (s), mean (SD)	11.46 (1.81)	11.23 (1.08)	0.37
aPTT (s), mean (SD)	25.9 (7.50)	23.7 (3.55)	0.02
MPV (fL), mean (SD)	8.75 (1.20)	9.20 (1.20)	0.07
Padua, mean (SD)	3.76 (2.48)	5.60 (2.10)	<0.01

* The higher standard deviation of the CRP than the mean is due to very high CRP value in some patients.

abs, absolute; aPTT, activated partial thromboplastin time; CRP, C-reactive protein; INR, international normalized ratio; LMWH, low-molecular-weight heparin; MPV, mean platelet volume; NOAC, non-vitamin K antagonist oral anticoagulant; PE, pulmonary embolism; PT, prothrombin time; SD, standard deviation.

A univariate logistic regression was performed. Ten variables were selected based on the likelihood-ratio test (Table 3).

**Table 3:** Selection of Variables by Logistic Regression.

Variable	LRT	LRT *P-*value	OR (95% CI)
Padua	14.0	<0.01	1.34 (1.14–1.58)
D-dimer	5.2	0.02	1.03 (1.00–1.06)
Platelet	5.9	0.02	0.995 (0.99–1.00)
aPTT	6.0	0.01	0.88 (0.78–0.98)
D-dimer/aPTT	8.9	<0.01	1.12 (1.03–1.20)
D-dimer/platelets	4.45	0.03	1.02 (0.99–1.06)
PLR	8.6	<0.01	0.68 (0.49–0.92)
PaDd	11.8	<0.01	1.01 (1.00–1.02)
PaDd/aPTT	18.1	<0.01	1.03 (1.02–1.05)
PaDd/platelets	8.4	<0.01	1.09 (1.01–1.19)

aPTT, activated partial thromboplastin time; CI, confidence interval; LRT, likelihood ratio test; OR, odds ratio; PaDd, Padua × D-dimer; PLR, platelet-to-lymphocyte ratio.

**Figure 2:**
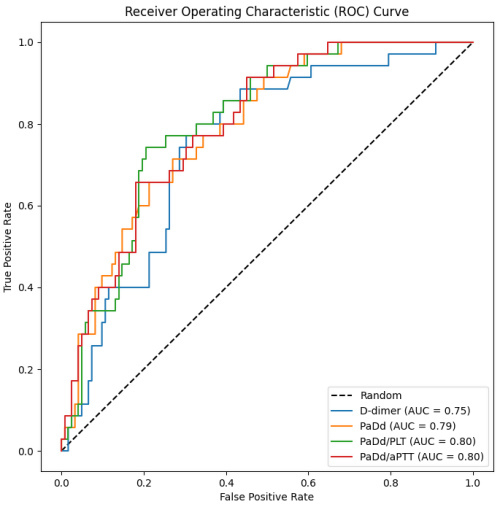
Receiver Operating Characteristic (ROC) Curves of D-dimer, PaDd, PaDd/PLT, and PaDd/aPTT. aPTT, activated partial thromboplastin time; AUC, area under the curve; PaDd, Padua × D-dimer; PLT, platelets.

Based on the univariate analysis, six variables were identified with an area under the curve of more than 0.7 (*P*<0.05) (Table 4); all of them contained Padua or D-dimer. Figure 2 provides the receiver operating characteristic curves for D-dimer, PaDd, PaDd/PLT, and PaDd/aPTT.

**Table 4:** Cut-off Independent Variables for PE Prediction with 100% Sensitivity.

Variable	Cutoff	Specificity	*P-*value	PPV
D-dimer	>1.23	0.09	<0.01	0.23
D-dimer/aPTT	>0.67	0.16	<0.01	0.25
D-dimer/platelets	>0.85	0.16	<0.01	0.29
PaDd	>5.93	0.32	<0.01	0.29
PaDd/platelets	>0.25	0.32	<0.01	0.30
PaDd/aPTT	>2.49	0.35	<0.01	0.31

aPTT, activated partial thromboplastin time; PaDd, Padua × D-dimer; PLR, platelet-to-lymphocyte ratio; PPV, positive predictive value.

The Padua score alone, platelets, aPTT, the platelet-to-lymphocyte ratio, and the D-dimer-to-platelet ratio were not retained because of the low area under the curve. More information about these rejected markers is provided in the supplement

## DISCUSSION

D-dimer levels are commonly interpreted with an age-adjusted threshold (age × 10) in elderly patients.^13^ Our study demonstrated that combining D-dimer levels with the Padua score (PaDd) enhances the identification of PE in individuals over 65 years, achieving 100% sensitivity and improving specificity from 9% to 32%. Applying this approach to our cohort could have decreased the number of CTs from 157 to 117—a 25% reduction. D-dimer alone remains a highly sensitive marker for PE detection, but its low specificity often leads to overuse of imaging for diagnostic evaluation. By combining D-dimer with the Padua score, specificity is improved without compromising sensitivity (Table 4). Further refinement by incorporating the platelet count and aPTT—resulting in the PaDd/PLT and PaDd/aPTT ratios—improved sensitivity based on the Youden index (Table A in the supplement). Among these, only the PaDd/aPTT ratio achieved 100% sensitivity while also improving specificity.

Regarding the PaDd/PLT marker, it has been well established that lower platelet counts increase the risk of PE.^28^,^29^ However, caution is required when interpreting these findings in patients with hematologic disorders, such as myelodysplastic syndromes.

Regarding the PaDd/aPTT marker, activated partial thromboplastin time is a measure of the overall adequacy of the intrinsic plasma-clotting factors. Prolonged aPTT is associated with an increased risk of bleeding^30^ and is theoretically expected to reduce the risk of embolism. However, some studies have reported that a longer aPTT (within the normal range) may, paradoxically, be a risk factor for PE.^31^,^32^ The discrepancies between these findings and our study may be explained by differences in study populations, as previous studies included a broader range of patients (e.g. intensive care unit patients, non-elderly populations) compared to our cohort. Additionally, prolonged aPTT can be observed in patients receiving direct non-vitamin K antagonist oral anti-coagulants (NOACs)^33^ or in conditions such as liver failure.^34^

### Consideration of Subsegmental PE

Subsegmental PE cases were classified as negative in our analysis.^35^ Observational studies suggest that withholding anticoagulation may not significantly impact three-month thromboembolic risk.^36^,^37^ Additionally, the diagnosis of subsegmental PE is subject to substantial interobserver variability,^38^ making its classification challenging. In our study, based on Youden’s index (the threshold that maximizes the sensitivity and specificity), applying a D-dimer threshold of 4.18 led to classification of 50% of subsegmental PE cases as negative and 50% as suspected PE (Tables A and B in the supplement). When using PaDd/aPTT (threshold 13.34), 75% were classified as negative, whereas with application of PaDd/ PLT (threshold 1.3), all were classified as suspected PE. Ensuring 100% sensitivity required lowering D-dimer PaDd/aPTT and PaDd/PLT thresholds (Table 4). This adjustment resulted in all subsegmental PE cases being categorized as suspected PE, except for one case when using the PaDd/aPTT ratio. When optimized for sensitivity, this classification approach identified nearly all subsegmental PE cases, supporting the robustness of our methodological approach.

### Role of Inflammatory and Alternative Biomarkers

Incorporating common inflammatory markers did not significantly improve the diagnostic accuracy of D-dimer in our study (supplement). The D-dimer/ CRP ratio, previously suggested for distinguishing pneumonia from PE,^26^ did not enhance specificity as shown by Berwick et al.^39^ Similarly, the immune-inflammation index, while of potential prognostic interest,^25^ lacked diagnostic utility in our cohort. Kara et al. showed that the D-dimer/fibrinogen ratio exhibited higher specificity but lower sensitivity,^22^ but in our study this marker did not reach statistical significance. Neutrophil-to-lymphocyte and platelet-to-lymphocyte ratios, which have been associated with deep vein thrombosis^23^ and cerebral vein thrombosis,^40^ were not significantly correlated with PE risk in our study. However, these markers may still hold prognostic relevance.^41^ Platelet count approached statistical significance, aligning with studies suggesting a decrease in platelet levels preceding PE.^29^ Although meta-analyses support an association between higher mean platelet volume and acute PE,^42^ our study found limited utility for this marker, potentially due to the older population and heterogeneity in mean platelet volume determinants.^43^ Calcium levels, while prognostically significant in PE,^44^ did not demonstrate clear diagnostic utility. Likewise, international normalized ratio was not predictive of PE in warfarin-treated patients,^45^ though it retains prognostic value.^46^

This study highlighted the clinical value of combining D-dimer with the Padua score (PaDd) to enhance PE diagnosis in elderly patients. By improving specificity while maintaining 100% sensitivity, this approach significantly reduces unnecessary CT scans, optimizing resource utilization and minimizing patient exposure to radiation. Further refinement by incorporating aPTT (PaDd/aPTT) to preserve 100% sensitivity further improved diagnostic accuracy, though caution is needed in patients with hematologic or coagulation disorders. Our findings also underscore the challenges in diagnosing PE in older populations, where conventional clinical scores are less reliable. While inflammatory and alternative biomarkers showed limited diagnostic utility, platelet count trends aligned with prior research on thrombotic risk. The classification of subsegmental PE remains a debated issue, but our approach ensures near-complete detection when prioritizing sensitivity. Overall, these results support the integration of PaDd-based strategies into clinical practice to refine PE diagnosis in elderly patients and reduce reliance on imaging. Given the high volume of daily CTs, this reduction is meaningful in terms of resource allocation and patient safety.

## STUDY LIMITATIONS

This study had several limitations. The conventional D-dimer threshold adjustment (age × 10) is widely accepted,^13^ but our cohort’s minimum D-dimer level in PE-positive cases was 1.23, potentially excluding some patients (e.g. a 70-year-old with a D-dimer of 0.8 μg/mL). The Padua score was designed for thrombosis risk assessment rather than PE diagnosis, and its emphasis on reduced mobility may contribute to misclassification.^47^ Although no patients with a Padua score of 0 had PE in our study, such cases are theoretically possible. Our small sample size limits generalizability, and complete case analysis resulted in exclusion of a significant proportion of subjects. Anticoagulant use is another important confounding factor. Some patients in our cohort were already on NOACs for atrial fibrillation or prior venous thromboembolism, potentially lowering PE risk. Additionally, NOACs may influence aPTT values,^33^ which could affect biomarker performance. Other factors such as acute infections, malignancies, and renal disease were not systematically controlled. Finally, as a single-center, retrospective study, our findings require prospective multicenter validation to confirm generalizability.

## FUTURE DIRECTIONS

Future research should focus on refining patient selection for CT by integrating clinical scores and laboratory markers, as demonstrated in this study. The Geneva Risk Score for Venous Thromboembolism^48^ and inflammatory markers such as ferritin and interleukins warrant further investigation. Given the interplay between thrombosis and immune pathways, the role of B lymphocytes and neutrophil extracellular traps should be explored.^49^ Including drug history (antiplatelets, NOACs) and simple imaging modalities (X-ray, ultrasound) may further enhance risk stratification.

One practical application of these findings could involve an algorithmic approach to PE diagnosis by stratifying patients into three categories: high risk (requiring immediate CT), low risk (CT not needed), and intermediate risk (potential wait-and-see strategy). In hemodynamically stable patients without contraindications to anticoagulation, an empirical treatment strategy—initiating anticoagulation while addressing other causes of dyspnea (e.g. pulmonary edema, COPD exacerbation) and reassessing after 2–3 days—may optimize CT utilization. However, prospective validation of this strategy is required.

## CONCLUSION

Biomarkers play a crucial role in reducing unnecessary imaging. The Padua score multiplied by D-dimer (PaDd) is a simple yet effective tool that enhances specificity while maintaining 100% sensitivity, ultimately reducing CT utilization in elderly patients. Prospective, multicenter studies are needed to validate these findings and integrate them into routine clinical practice.

## Supplementary Information



## Abbreviations

aPTT: activated partial thromboplastin time
CRP: C-reactive protein
CT: computed tomography
INR: international normalized ratio
MPV: mean platelet volume
NOAC: non-vitamin K antagonist oral anti-coagulant
PE: pulmonary embolism.

## References

[1] Goldhaber SZ (1998). Pulmonary embolism. N Engl J Med.

[2] Andreucci M, Solomon R, Tasanarong A (2014). Side effects of radiographic contrast media: pathogenesis, risk factors, and prevention. Biomed Res Int.

[3] Henzler T, Barraza JM, Nance JW (2011). CT imaging of acute pulmonary embolism. J Cardiovasc Comput Tomogr.

[4] Berman AR (2001). Pulmonary embolism in the elderly. Clin Geriatr Med.

[5] Borders C, Sajjadi SA (2021). Diagnosis and management of cognitive concerns in the oldest-old. Curr Treat Options Neurol.

[6] Gardner RC, Valcour V, Yaffe K (2013). Dementia in the oldest old: a multi-factorial and growing public health issue. Alzheimers Res Ther.

[7] Pierce AL, Kawas CH (2017). Dementia in the oldest old: beyond Alzheimer disease. PLoS Med.

[8] Skinner TR, Scott IA, Martin JH (2016). Diagnostic errors in older patients: a systematic review of incidence and potential causes in seven prevalent diseases. Int J Gen Med.

[9] Chan TF, Ngian VJJ, Hsu K, Frankel A, Ong BS (2020). Pulmonary embolism: clinical presentation and diagnosis in the oldest old. Intern Med J.

[10] Klok FA, Kruisman E, Spaan J (2008). Comparison of the revised Geneva score with the Wells rule for assessing clinical probability of pulmonary embolism. J Thromb Haemost.

[11] Weitz JI, Fredenburgh JC, Eikelboom JW (2017). A test in context: D-dimer. J Am Coll Cardiol.

[12] Schutte T, Thijs A, Smulders YM (2016). Never ignore extremely elevated D-dimer levels: they are specific for serious illness. Neth J Med.

[13] Freund Y, Chauvin A, Jimenez S (2021). Effect of a diagnostic strategy using an elevated and age-adjusted D-dimer threshold on thromboembolic events in emergency department patients with suspected pulmonary embolism: a randomized clinical trial. JAMA.

[14] Takach Lapner S, Julian JA, Linkins LA, Bates S, Kearon C (2017). Comparison of clinical probability-adjusted D-dimer and age-adjusted D-dimer interpretation to exclude venous thromboembolism. Thromb Haemost.

[15] Zhang K, Zhu Y, Tian Y, Tian M, Li X, Zhang Y (2021). Role of a new age-adjusted D-dimer cutoff value for preoperative deep venous thrombosis exclusion in elderly patients with hip fractures. J Orthop Surg Res.

[16] Ahmed YAY, Elfadl AEAA, Houssein A, Abdelrahman AA, Shehata KMA, Omar AOM (2024). Prognostic value of laboratory markers in patients with acute pulmonary embolism. Research Square [Preprint].

[17] Kundi H, Balun A, Cicekcioglu H (2015). The relation between platelet-to-lymphocyte ratio and Pulmonary Embolism Severity Index in acute pulmonary embolism. Heart Lung.

[18] Ozcan Cetin EH, Cetin MS, Canpolat U (2017). Platelet-to-lymphocyte ratio as a novel marker of in-hospital and long-term adverse outcomes among patients with acute pulmonary embolism: a single center large-scale study. Thromb Res.

[19] Zhou H, Hu Y, Li X (2018). Assessment of the risk of venous thromboembolism in medical inpatients using the Padua prediction score and Caprini risk assessment model. J Atheroscler Thromb.

[20] Kandagatla P, Goranta S, Antoine H, Marashi SM, Schmoekel N, Gupta AH (2019). PADUA score as a predictor for pulmonary embolism: a potential strategy for reducing unnecessary imaging. J Thromb Thrombolysis.

[21] Wikan VE, Tøndel BG, Morelli VM, Brodin EE, Brækkan SK, Hansen JB (2023). Diagnostic blood biomarkers for acute pulmonary embolism: a systematic review. Diagnostics (Basel).

[22] Kara H, Bayir A, Degirmenci S (2014). D-dimer and D-dimer/fibrinogen ratio in predicting pulmonary embolism in patients evaluated in a hospital emergency department. Acta Clin Belg.

[23] Selvaggio S, Brugaletta G, Abate A (2023). Platelet-to-lymphocyte ratio, neutrophil-to-lymphocyte ratio and monocyte-to-HDL cholesterol ratio as helpful biomarkers for patients hospitalized for deep vein thrombosis. Int J Mol Med.

[24] Xue J, Ma D, Jiang J, Liu Y (2021). Diagnostic and prognostic value of immune/inflammation biomarkers for venous thromboembolism: is it reliable for clinical practice?. J Inflamm Res.

[25] Seo J, Cho JH, Ohk TG, Lee HY, Park CW (2021). Use of ratio of D-dimer to C-reactive protein as an adjunctive method to differentiate between pulmonary embolism and pneumonia in elderly patients. J Korean Soc Emerg Med.

[26] Woodward M, Lowe GDO, Campbell DJ (2005). Associations of inflammatory and hemostatic variables with the risk of recurrent stroke. Stroke.

[27] Tanashat M, Moawad MHED, Ramadan A (2023). Abstract 14433: The utility of fibrinogen-albumin-ratio as a prognostic biomarker in coronary artery disease patients: a comprehensive meta-analysis. Circulation.

[28] Monreal M, Lafoz E, Casals A, Ruíz J, Arias A (1991). Platelet count and venous thromboembolism. A useful test for suspected pulmonary embolism. Chest.

[29] Kovács S, Csiki Z, Zsóri KS, Bereczky Z, Shemirani AH (2019). Characteristics of platelet count and size and diagnostic accuracy of mean platelet volume in patients with venous thromboembolism. A systematic review and meta-analysis. Platelets.

[30] Proctor RR, Rapaport SI (1961). The partial thromboplastin time with kaolin. A simple screening test for first stage plasma clotting factor deficiencies. Am J Clin Pathol.

[31] Huang CB, Hong CX, Xu TH (2022). Risk factors for pulmonary embolism in ICU patients: a retrospective cohort study from the MIMIC-III database. Clin Appl Thromb Hemost.

[32] Zhou Q, Xiong XY, Liang ZA (2022). Developing a nomogram-based scoring tool to estimate the risk of pulmonary embolism. Int J Gen Med.

[33] Samuelson BT, Cuker A, Siegal DM, Crowther M, Garcia DA (2017). Laboratory assessment of the anticoagulant activity of direct oral anticoagulants: a systematic review. Chest.

[34] Verhaeghe R, van Damme B, Molla A, Vermylen J (1972). Dysfibrinogenaemia associated with primary hepatoma. Scand J Haematol.

[35] Goy J, Lee J, Levine O, Chaudhry S, Crowther M (2015). Sub-segmental pulmonary embolism in three academic teaching hospitals: a review of management and outcomes. J Thromb Haemost.

[36] Mehta D, Barnett M, Zhou L (2014). Management and outcomes of single subsegmental pulmonary embolus: a retrospective audit at North Shore Hospital, New Zealand. Intern Med J.

[37] Carrier M, Righini M, Wells PS (2010). Subsegmental pulmonary embolism diagnosed by computed tomography: incidence and clinical implications. A systematic review and meta-analysis of the management outcome studies. J Thromb Haemost.

[38] Gupta A, Raja AS, Ip IK, Khorasani R (2014). Assessing 2 D-dimer age-adjustment strategies to optimize computed tomographic use in ED evaluation of pulmonary embolism. Am J Emerg Med.

[39] Berwick R, Navalkissoor S, Hurst JR (2011). P8 Use of D-dimer: CRP ratio compared to D-dimer alone to predict PE on VQ scanning. Thorax.

[40] Artoni A, Abbattista M, Bucciarelli P (2018). Platelet to lymphocyte ratio and neutrophil to lymphocyte ratio as risk factors for venous thrombosis. Clin Appl Thromb Hemost.

[41] Phan T, Brailovsky Y, Fareed J, Hoppensteadt D, Iqbal O, Darki A (2020). Neutrophil-to-lymphocyte and platelet-to-lymphocyte ratios predict all-cause mortality in acute pulmonary embolism. Clin Appl Thromb Hemost.

[42] Febra C, Macedo A (2020). Diagnostic role of mean-platelet volume in acute pulmonary embolism: a meta-analysis and systematic review. Clin Med Insights Circ Respir Pulm Med.

[43] Korniluk A, Koper-Lenkiewicz OM, Kamińska J, Kemona H, Dymicka-Piekarska V (2019). Mean platelet volume (MPV): new perspectives for an old marker in the course and prognosis of inflammatory conditions. Mediators Inflamm.

[44] Wang X, Xiang Y, Zhang T, Yang Y, Sun X, Shi J (2020). Association between serum calcium and prognosis in patients with acute pulmonary embolism and the optimization of pulmonary embolism severity index. Respir Res.

[45] Hansen P, Zmistowski B, Restrepo C, Parvizi J, Rothman RH (2012). Does international normalized ratio level predict pulmonary embolism?. Clin Orthop Relat Res.

[46] Kırış T, Yazıcı S, Durmuş G (2018). The relation between international normalized ratio and mortality in acute pulmonary embolism: a retrospective study. J Clin Lab Anal.

[47] Ye F, Bell LN, Mazza J, Lee A, Yale SH (2018). Variation in definitions of immobility in pharmacological thromboprophylaxis clinical trials in medical inpatients: a systematic review. Clin Appl Thromb Hemost.

[48] Fu Z, Zhuang X, He Y, Huang H, Guo W (2020). The diagnostic value of D-dimer with simplified Geneva score (SGS) pre-test in the diagnosis of pulmonary embolism (PE). J Cardiothorac Surg.

[49] Hasselwander S, Xia N, Mimmler M (2022). B lymphocyte-deficiency in mice promotes venous thrombosis. Heliyon.

