# Supplementary material for: Padua Score and D-dimer for Pulmonary Embolism Exclusion in the Elderly
Source: Rambam Maimonides Med J. 2025 Jul 31;16(3):e0013. doi: 10.5041/RMMJ.10548 (PMC12316886; doi:10.5041/RMMJ.10548)
Supplement: Supplementary file 1 [file rmmj-16-3-e0013_supplement.pdf]

SUPPLEMENTARY MATERIAL

This appendix has been provided by the authors for the benefit of readers

Supplement to Padua Score and D-dimer for Pulmonary Embolism Exclusion in the Elderly

Cohen R, Nemet S, Fradkin MA, Schiller T, Kirzhner A, Abu Khadija H, Deitch M, Elbirt D. Padua Score and D-dimer for Pulmonary Embolism Exclusion in the Elderly. Rambam Maimonides Med J 2025;16 (3):e0013. doi:10.5041/RMMJ.10548

Table A. Performance Statistics of Variables for Predicting Pulmonary Embolism.

Variable	Cutoff	Sensitivity	Specificity	Youden's Index	AUC (95% CI)	*P*-value
D-dimer	>4.18	0.8	0.67	0.47	0.75 (0.64-0.83)	<0.01
D-dimer/aPTT	>1.61	0.82	0.64	0.47	0.76 (0.66-0.83)	<0.01
D-dimer/platelet	>1.57	0.86	0.59	0.48	0.76 (0.67-0.82)	0.02
PaDd	>29.26	0.65	0.79	0.44	0.79 (0.69-0.86)	<0.01
PaDd/platelet	>1.3	0.74	0.80	0.54	0.80 (0.70-0.86)	<0.01
PaDd/aPTT	>13.34	0.66	0.82	0.48	0.80 (0.70-0.86)	<0.01

aPTT, activated partial thromboplastin time; AUC, area under the curve; CI, confidence interval; PaDd, Padua × D-dimer.

Table B. Sub-segmental Data of Pulmonary Embolism Patients.

Patient	D-dimer (µg/mL)	Sub-segmental Data				
		D-dimer/aPTT	D-dimer/Platelets	PaDd	PaDd/Platelets	PaDd/aPTT
1	14.07	5.80	7.56	14.07	0.75	5.81
2	46.71	13.01	17.90	233.55	8.98	65.05
3	1.44	0.60	0.55	10.08	0.38	4.20
4	76.19	26.36	33.70	76.19	113.75	26.36
5	1.75	0.76	0.76	5.25	0.23	2.29
6	3.94	1.97	1.03	19.70	0.51	9.85
7	3.00	1.11	1.40	18.00	0.84	6.69
8	25.63	16.01	7.54	102.00	3.01	64.00

aPTT, activated partial thromboplastin time; PaDd, Padua × D-dimer.

Figure A. Receiver Operating Characteristic Curves for All Variables.

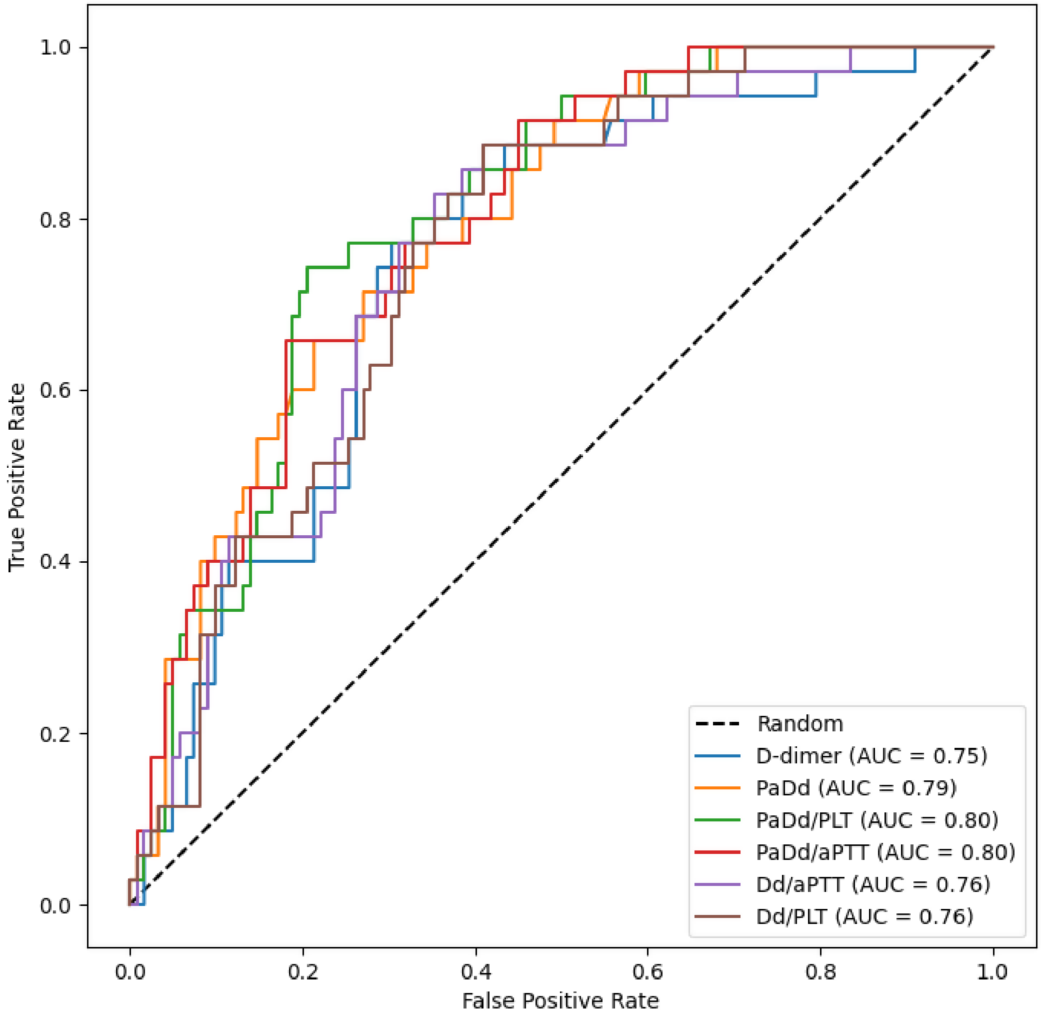

Data for all variables are provided on the following pages.

ALL VARIABLES

Results for Variable: Padua
- Likelihood ratio test (LRT) statistic: 14.06
- Likelihood ratio test (LRT) *P*-value: 0.000
- Odds ratio: 1.34
- 95% Confidence interval: [1.14, 1.58]

Results for Variable: Age
- LRT statistic: 2.47
- LRT *P*-value: 0.116
- Odds ratio: 1.03
- 95% Confidence interval: [0.99, 1.08]

Results for Variable: D-dimer
- LRT statistic: 5.18
- LRT *P*-value: 0.023
- Odds ratio: 1.03
- 95% Confidence interval: [1.00, 1.06]

Results for Variable: Fibrinogen
- LRT statistic: 0.98
- LRT *P*-value: 0.322
- Odds ratio: 1.00
- 95% Confidence interval: [1.00, 1.00]

Results for Variable: Platelet
- LRT statistic: 5.86
- LRT *P*-value: 0.016
- Odds ratio: 1.00
- 95% Confidence interval: [0.99, 1.00]

Results for Variable: Neutrophil Absolute (abs)
- LRT statistic: 0.15
- LRT *P*-value: 0.696
- Odds ratio: 1.02
- 95% Confidence interval: [0.93, 1.11]

Results for Variable: Lymphocyte Absolute (abs)
- LRT statistic: 2.51
- LRT *P*-value: 0.113
- Odds ratio: 1.39
- 95% Confidence interval: [0.92, 2.09]

Results for Variable: CRP
- LRT statistic: 0.03
- LRT *P*-value: 0.857
- Odds ratio: 1.00
- 95% Confidence interval: [0.95, 1.04]

Results for Variable: Albumin
- LRT statistic: 0.57
- LRT *P*-value: 0.450
- Odds ratio: 0.72
- 95% Confidence interval: [0.31, 1.67]

Results for Variable: Calcium
- LRT statistic: 1.10
- LRT *P*-value: 0.295
- Odds ratio: 1.31
- 95% Confidence interval: [0.79, 2.16]

Results for Variable: INR
- LRT statistic: 0.96
- LRT *P*-value: 0.327
- Odds ratio: 0.47
- 95% Confidence interval: [0.05, 4.63]

Results for Variable: PT
- LRT statistic: 0.53
- LRT *P*-value: 0.468
- Odds ratio: 0.91
- 95% Confidence interval: [0.70, 1.19]

Results for Variable: aPTT
- LRT statistic: 6.09
- LRT *P*-value: 0.014
- Odds ratio: 0.88
- 95% Confidence interval: [0.78, 0.98]

Results for Variable: MPV
- LRT statistic: 3.13
- LRT *P*-value: 0.077
- Odds ratio: 1.32
- 95% Confidence interval: [0.97, 1.79]

Results for Variable: D-Dimer/Platelet
- LRT statistic: 4.45
- LRT *P*-value: 0.035
- Odds ratio: 1.03
- 95% Confidence interval: [0.99, 1.06]

Results for Variable: Padua/Platelet
- LRT statistic: 8.42
- LRT *P*-value: 0.004
- Odds ratio: 1.09
- 95% Confidence interval: [1.01, 1.19]

Results for Variable: Neutrophil/ Lymphocyte
- LRT Statistic: 1.80
- LRT *P*-value: 0.180
- Odds ratio: 1.00
- 95% Confidence interval: [1.00, 1.01]

Results for Variable: D-Dimer/aPTT
- LRT statistic: 8.99
- LRT *P*-value: 0.003
- Odds ratio: 1.12
- 95% Confidence interval: [1.04, 1.20]

Results for Variable: Padua/aPTT
- LRT statistic: 18.14
- LRT *P*-value: <0.001
- Odds ratio: 1.03
- 95% Confidence interval: [1.02, 1.05]
